# Impact of orally administered lozenges with *Lactobacillus rhamnosus* GG and *Bifidobacterium animalis* subsp. *lactis* BB-12 on the number of salivary mutans streptococci, amount of plaque, gingival inflammation and the oral microbiome in healthy adults

**DOI:** 10.1007/s00784-014-1221-6

**Published:** 2014-03-18

**Authors:** Aino Toiviainen, Heli Jalasvuori, Emilia Lahti, Ulvi Gursoy, Seppo Salminen, Margherita Fontana, Susan Flannagan, George Eckert, Alexis Kokaras, Bruce Paster, Eva Söderling

**Affiliations:** 1 Institute of Dentistry, University of Turku, Lemminkäisenkatu 2, 20520 Turku, Finland; 2 Functional Foods Forum, University of Turku, Itäinen Pitkäkatu 4 A, 20520 Turku, Finland; 3 Department of Cariology, Restorative Sciences & Endodontics, School of Dentistry, University of Michigan, 1011 N University Ave, Ann Arbor, MI 48109 USA; 4 Department of Microbiology, The Forsyth Institute, 245 1st St, Cambridge, MA 02142 USA; 5 Department of Oral Medicine, Infection & Immunity, Harvard School of Dental Medicine, Boston, MA 02115 USA

**Keywords:** *Lactobacillus rhamnosus* GG, *Bifidobacterium lactis* BB-12, Probiotic, HOMIM, Mutans streptococci, Lactobacilli, Plaque index, Gingival index

## Abstract

**Objectives:**

The aim was to evaluate the effects of orally administered *Lactobacillus rhamnosus* GG (LGG) and *Bifidobacterium animalis* subsp. *lactis* BB-12 (BB-12) on the number of salivary mutans streptococci (MS), amount of plaque, gingival inflammation and the oral microbiota in healthy young adults.

**Materials and methods:**

The study was a randomised, controlled, double-blind trial. Healthy volunteers used lozenges containing a combination of LGG and BB-12 (test group, *n* = 29) or lozenges without added probiotics (control group, *n* = 31) for 4 weeks. At baseline and at the end of the test period, the plaque index (PI) and gingival index (GI) were determined, and stimulated saliva was collected. The microbial composition of saliva was assessed using human oral microbe identification microarray (*n* = 30). MS and lactobacilli (LB) were plate cultured.

**Results:**

The probiotic lozenge decreased both PI and GI (*p* < 0.05) while no changes were observed in the control group. However, no probiotic-induced changes were found in the microbial compositions of saliva in either group.

**Conclusions:**

The probiotic lozenge improved the periodontal status without affecting the oral microbiota.

**Clinical relevance:**

Short-term consumption of LGG and BB-12 decreased the amount of plaque which was associated with a clinical impact: a decrease in gingival inflammation.

## Introduction

Recent studies have demonstrated the health impact of specific probiotic bacteria for humans and several recommendations for probiotic use have appeared. The most widely studied species belong to the genera *Lactobacillus* and *Bifidobacterium. Lactobacillus rhamnosus* GG (LGG) has been reported to be effective in preventing and treating rotavirus diarrhea, atopic eczema and upper respiratory infections [1–3]. The most thoroughly investigated *Bifidobacterium* strain *Bifidobacterium lactis* BB-12 (BB-12) has been used in prevention/treatment of diarrhea and respiratory infections [4, 5]. In addition, the combination of these two probiotics appears to possess additional efficacy in prevention and treatment of allergic disorders, acute respiratory infections and acute otitis media [6–8]. Recently, the stability of probiotics has been reported to be improved by enhancing the tolerance to processing conditions, and both *L. rhamnosus* GG and *B. lactis* could be improved without altering their basic probiotic properties [9, 10].

Most probiotics colonise the gut only temporarily [11]. Thus, to obtain health benefits from consuming probiotics, the consumption should take place on a daily basis. From a dental point of view, however, acidogenic and aciduric lactobacilli and bifidobacteria are generally considered cariogenic [12] and could be regarded as a risk for dental health. Even though some probiotics like LGG and *Lactobacillus reuteri* can form biofilm [13, 14], most studies suggest that intake of probiotics leads only to a transient oral colonisation [15–18]. Furthermore, the existing clinical studies suggest that probiotics are safe for teeth and they may even have beneficial effects on dental health [19–23].

There are several mechanisms by which probiotics may influence oral health, e.g. immune modulation, impact on the oral microbiome, production of antimicrobial substances by the probiotic or the modified microbiome, competitive exclusion and hindering adhesion of oral pathogens [24]. All available data demonstrate that effects of probiotics are both species and strain specific. Most studies on probiotics and oral health have focused on measuring changes in mutans streptococci (MS) counts [25–27]. Even though high counts of MS do not necessarily mean an increased caries risk, decreasing MS without affecting the microbiota should make the plaque less virulent. Several studies have suggested that *L. reuteri* decreases counts of MS [27] even though also contradictory results have been published [28]. LGG consumption lasting from 2 weeks to 7 months has been associated with decreases or no changes in MS counts [19, 28, 29]. Short-term *B. lactis* consumption showed decreases in MS counts [27]. However, apart from MS, very little is known about effects of probiotics on the microbiota. Effects of probiotics on periodontal pathogens has received little interest so far, even though, administration of some probiotics has been associated with improved gingival and periodontal conditions [24].

Based on the health benefits reported for the combination of LGG and BB-12, we decided to assess the impact of this probiotic combination on plaque accumulation, gingival health and the oral microbiota in healthy subjects. The null hypothesis was that the consumption of the LGG-BB-12 combination has no effect on the number of salivary MS, plaque index, gingival index or oral microbiota.

## Materials and methods

### Subjects

Altogether 77 volunteer students of the University of Turku, Finland, were screened for the presence of salivary MS (Dentocult® SM Strip Mutans, Orion Diagnostica, Espoo, Finland). The inclusion criteria were good general health, willingness to participate and salivary MS counts ≥10^3^ colony-forming units (CFU)/ml. The 62 subjects showing salivary MS counts of ≥10^3^ CFU/ml were invited to participate and they volunteered for the study. The study was approved by the ethics committee of the Hospital District of Southwest Finland: ETMK 22/180/2012. A written informed consent was obtained from all subjects. The ethics committee defined the stopping rules. The ClinicalTrials.gov identifier of the study is NCT01577485.

Subjects were randomly divided into two groups: group 1 with a mean age (SD) of 24.6 (2.7) years (3 males, 28 females), and group 2 with a mean age (SD) of 24.0 (3.0) years (4 males, 27 females). Fifty-nine subjects had normal flow rates of paraffin-stimulated saliva (>1 ml/min); three subjects had flow rates <1 ml/min (1/group 1 and 2/group 2). All subjects had good oral hygiene with no immediate need for major restorative dentistry.

None of the subjects smoked. All subjects in group 2 (control group) completed the study. In group 1 (probiotic group), one female subject dropped out during the wash-out period, and another female subject dropped out during the test period. Both subjects reported gastrointestinal problems as the reason for dropping out.

### Study design

The study was a randomised, controlled, double-blind trial. A 4-week run-in period was chosen based on earlier studies [15–17]. At the beginning of the run-in period, the subjects were given written instructions as follows: commercial products containing *L. rhamnosus* GG or *B. lactis* BB-12 were not allowed but otherwise the subjects were to maintain their normal tooth brushing and dietary habits. None of the subjects were habitual consumers of such products before the run-in period. All subjects used xylitol products (mostly chewing gum) on daily basis before the study. The subjects were instructed not to use their regular xylitol products during the study. Most subjects brushed their teeth twice a day. Throughout the study, the subjects used the same brand of fluoridated toothpaste provided to them free of charge. The drinking water in the city of Turku contains 0.3 mg/l fluoride. Antimicrobial mouthrinses were not allowed. Two of the subjects had been on a brief course of antimicrobial medication 3 weeks before the study started, and one subject used low-concentration tetracycline medication throughout the study. Compliance to given instructions was checked at both sampling visits (baseline and 4 weeks after). At these visits, the subjects were also interviewed for dietary habits and oral/general health. These data were used to check for compliance, possible adverse effects and other confounding factors.

The subjects were randomly allocated into two groups (groups 1 and 2): both groups used the same run-in chewing gum for 4 weeks before the test period started. The recommendation for the use of the run-in gum was 4 pieces per day. After the run-in, group 1 used green-coded test lozenges and group 2 used yellow-coded control lozenges for 4 weeks. Also for the lozenges, the recommendation was 4 pieces per day. The recommended number of gums/lozenges resulted in a daily xylitol dose of approximately 2 g which, according to several studies, is too low to exert any “xylitol-effects” on the oral microbiota [30]. Since most of the subject regularly consumed xylitol prior to the study, to ensure compliance both the wash-out chewing gum and test lozenge contained xylitol as one of the bulk agents. The daily amount of probiotics was approximately 2 × 10^9^ cells for LGG and BB-12 each. The first of four lozenges was instructed to be taken in the morning, and the last one in the evening following brushing of the teeth. All but one of the subjects, who used only two lozenges per day, reported using four lozenges per day. The subjects were asked to chew the lozenge thoroughly to disrupt its structure before swallowing.

### Test products

The run-in chewing gum was a mild tasting, noncommercial chewing gum manufactured free of charge for the study by Karl Fazer AB (Vantaa, Finland). The gum pieces weighed 1.2 g and contained 42 % xylitol, 18 % sorbitol and 5 % maltitol. The 1-g lozenges used during the test period were also manufactured by Fazer. They were compressed from 50 % xylitol and 46 % sorbitol. The *L. rhamnosus* GG (ATCC 53103) used in the probiotic lozenges was from Probiotical S.p.A., Novara, Italy. The *B. lactis* BB-12® (DSM15954) was from Chr. Hansen A/S, Hoersholm, Denmark. Each probiotic lozenge contained LGG 4.4 × 10^8^ and BB-12 4.8 × 10^8^. The stability of the probiotics was improved before tableting as described earlier [9], thus the viability of the probiotics did not change during manufacturing or storage at room temperature. The control lozenge had the same polyol composition but contained no probiotics. The appearance and solubility properties of the probiotic lozenge were similar to that of the control lozenge with no probiotics. The chewing gums and lozenges were supplied in plastic bottles containing adsorbent pads to control humidity. The control/test lozenge bottles were marked with a yellow/green sticker. A 4.5-week supply was given to all subjects, along with instructions to store the bottles at room temperature.

### Outcome measures, sample size and blinding

The primary outcome measure was the MS level in saliva and the secondary outcome measure the plaque index. As demonstrated in an earlier study, a significant probiotic-induced decrease in MS levels could be detected with as few as 10 MS-positive subjects; thus, sample size calculation was not used here [31]. Furthermore, since we could compare the baseline MS levels with the values obtained after the consumption of the control/probiotic lozenge, paired sample analysis, sensitive for even small changes in the variables, could be used.

The second and third authors (HJ, EL), who recruited the subjects and collected all samples, as well as the technical assistants analysing the samples, were blinded to the study assignment. The group codes were revealed to the researchers only after the statistical analyses had been performed.

### Determination of plaque and gingival index

Plaque was allowed to accumulate for 24 h with no oral hygiene at baseline and at the end of each tablet period. On the morning of the plaque collection day, the subjects were instructed not to use the run-in chewing gum/test tablet. The Silness-Löe plaque index (PI; [32]) and the Löe-Silness gingival index (GI; [33]) were determined in the afternoon using a periodontal probe. Care was taken to avoid bleeding.

### Saliva collection for human oral microbe identification microarray analyses and plate culturing

Following the determination of PI and GI, 4 ml paraffin-stimulated saliva was collected and the collection time was recorded. For the human oral microbe identification microarray (HOMIM) analyses, the saliva samples of 15 randomly chosen subjects per group were selected. One milliliter samples of the saliva were pipetted onto 10 μl TE buffer (Sigma-Aldrich, St. Louis, MO, USA) and stored at −70 °C before shipment on dry ice. Genomic DNA was purified using MasterPure Gram Positive DNA Purification kit (Epidentre Biotechnologies, Madison, WI, USA), with modifications (http://mim.forsyth.org). For plate culturing, samples (100 μl) of the saliva were added into 900 μl Tryptic Soy Broth with 10 % glycerol (TSB; Scharlau Chemie S.A., Barcelona, Spain) and stored at −70 °C before microbial analyses.

### HOMIM

At the Forsyth Institute, microbial profiles were generated from image files of scanned HOMIM microarrays (http://bioinformatics.forsyth.org/homim/). In brief, concentration levels of approximately 300 oral taxa were determined by microarray hybridisation using a fluorescent readout reverse-capture method [34]. Fluorescently labelled sample microbial DNA was captured by 16S rRNA-based probes attached to glass slides. The fluorescent intensity for each probe was scanned, normalised and scaled as previously reported [34]. Signals of 2× background were considered to be negative and assigned a HOMIM level score of 0. Positive hybridisation signals were categorised into five levels, with 1 indicating a signal that was just detectable, and 5 indicating maximum signal intensity.

### Plate culturing of MS and lactobacilli

The TSB tubes were thawed and vortexed for 1 min. The content was pumped back-and-forth using a disposable pipette and the resulting suspension was mildly sonicated. After serial tenfold dilutions, the bacteria were plated on Difco^TM^ Mitis salivarius (Becton Dickinson and Company, Sparks, MD, USA) agar containing bacitracin (MSB). The plates were incubated at +37 °C in a 7 % CO_2_ atmosphere for 2 days. MS were identified as described earlier [35]. The number of MS was identified based on colony morphology and counted by means of a stereomicroscope. The identification of *Streptococcus mutans* was based on consistent findings of “rough” colony morphology; positive fermentation with sorbitol, mannitol, raffinose and melibiose; and negative dextran agglutination. Identification of *Streptococcus sobrinus* was based on “smooth” colonies, positive fermentation with mannitol but negative with raffinose and melibiose, and positive dextran agglutination. The lactobacilli and were grown for 2 days anaerobically (80 % N_2_, 10 % CO_2_, 10 % H_2_) at +37 °C on Difco^TM^ Rogosa SL agar (Becton Dickinson and Company). The results were expressed as colony-forming units per millilitre. The detection limit of the plate culturing was 100 CFU/ml.

### Plaque collection and determination of the polysaccharide content of the plaque samples

After the saliva collection, all available supragingival plaque was collected with dental curettes from the left half of the mouth and suspended in tubes containing 0.5 ml 1 N NaOH. The plaque samples were stored at −70 °C before analyses. The sugars of hydrolysed plaque reflect the polysaccharide content of plaque and the proteins reflect the levels of microbes in the plaque [36, 37]. For determining the sugar/protein ratio, the test tubes with the plaque samples in NaOH were heated for 1 hour in a boiling water bath. Thereafter, sugar [38] and protein [39] concentrations were determined and their ratio was calculated.

### Statistical analysis

*t* test for paired samples and Wilcoxon signed ranks test were used to study differences in PIs, GIs and salivary MS and lactobacilli counts between baseline and the end of the test period. Independent samples *t* test was used to compare values between the two groups at baseline and after the test period. Pearson’s test was used to calculate the correlations. A multinomial logistic regression analysis was performed to study the associations of the GI and PI in the study groups. The program package SPSS 19.0 for Windows was used. The level of statistical significance was set at *p* < 0.05.

The HOMIM results were analysed at the University of Michigan. Change from pretreatment to post-treatment was tested within each group using Wilcoxon signed rank tests. Comparisons between groups for differences pretreatment and for differences in the change from pretreatment to post-treatment were made using Wilcoxon rank sum tests. To control the overall level of significance for the large number of tests in the HOMIM analyses, *p* values were evaluated using an false discovery rate (FDR) adjustment. SAS version 9.3 was used for these analyses.

## Results

The mean PI and GI values decreased significantly in the probiotic group while no change was observed in the control group (*p* = 0.016 for PI and *p* = 0.012 for GI; Table 1). The number of cases with decreased PI and GI values in the probiotic group showed a positive correlation (*r* = 0.389; *p* = 0.037) and they were also significantly associated (OR 5.8, 95 % CI 1.0–30). In the control group, the number of subjects with decreased PI and GI values did not correlate and no association was found.

**Table 1:** Impact of probiotic treatment on plaque and gingival indices at baseline and 4 weeks after the use of the LGG + BB12/control lozenge. Means ± SD given

	Plaque index			Gingival index		
Group	Baseline	4 weeks	*p*	Baseline	4 weeks	*p*
Control (*n* = 31)	0.94 ± 0.40	0.82 ± 0.35	n.s.	0.70 ± 0.44	0.61 ± 0.37	n.s.
LGG + BB-12 (*n* = 29)	1.10 ± 0.36	0.94 ± 0.35	0.016	0.71 ± 0.28	0.57 ± 0.23	0.012

**Fig. 1:**
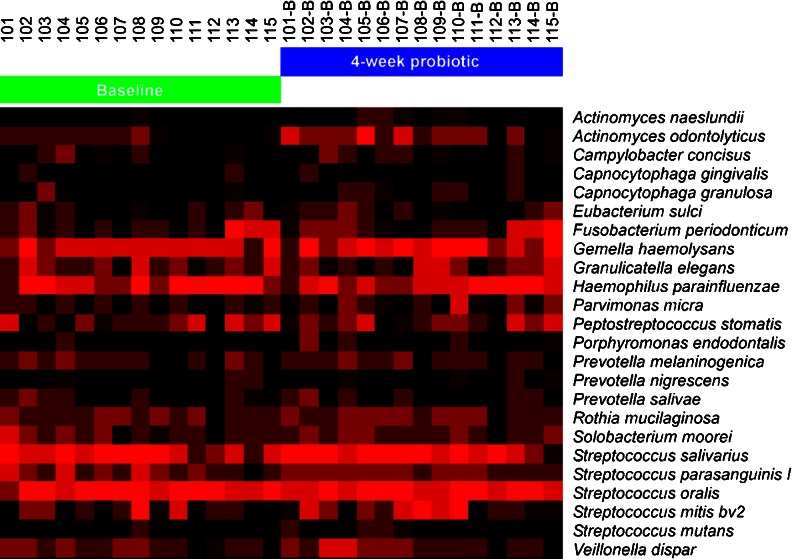
Individual HOMIM profiles for 27 selected predominant microbes representing the oral microbiota of the 15 subjects of the probiotic group at baseline and after 4-week use of the probiotic lozenge. The color intensity reflects relative proportions of the species present in the sample

The HOMIM analysis included probes for about 300 predominant oral bacterial taxa. According to the results, microbiota at baseline did not differ between the groups, and none of the subjects harboured *Porphyromonas gingivalis*, *Prevotella intermedia* or *Aggregatibacter actinomycetemcomitans*. Only three subjects had lactobacilli present in their saliva. After the FDR adjustment, the analyses did not reveal any statistically significant changes in the microbiota when baseline values were compared with values obtained after the test period. Figure 1 shows individual HOMIM profiles for 27 selected predominant microbes representing the oral microbiota of the subjects of the probiotic group at baseline and after 4-week use of the probiotic lozenge.

At baseline, all subjects harboured *S. mutans* in their saliva. Of these, 14 subjects in the probiotic group and 19 subjects in the control group had high salivary MS counts (>10^5^ CFU/ml). Only one subject showed a combination of *S. mutans* and *S. sobrinus*; thus, results for mutans streptococci (MS) are shown. No study-induced changes in the MS counts were detected either in the probiotic or the control group (Table 2). At baseline, 15 subjects in the probiotic group and 14 subjects in the control group had detectable levels of lactobacilli in their saliva. The use of the LGG-containing lozenges was not reflected in the salivary lactobacilli counts of the subjects. No changes were detected in the counts of lactobacilli when baseline values were compared with the values obtained after the test period.

**Table 2:** Counts of mutans streptococci and lactobacilli (log CFU/ml saliva) at baseline and 4 weeks after the use of the LGG + BB12/control lozenge. Means ± SD given

	Mutans streptococci			Lactobacilli		
Group	Baseline	4 weeks	*p*	Baseline	4 weeks	*p*
Control (*n* = 31)	4.22 ± 1.77	4.42 ± 1.61	n.s.	1.74 ± 2.04	2.47 ± 2.09	n.s.
LGG + BB-12 (*n* = 29)	4.63 ± 0.69	4.55 ± 0.91	n.s.	2.14 ± 2.18	2.50 ± 2.24	n.s.

The polysaccharide content of plaque may reflect plaque adhesion and virulence. No study-induced differences were detected in the sugar/protein ratios of the plaques (data not shown).

Only two subjects dropped out of the study: one during the wash-out period and one during the test period. Both reported that gastrointestinal problems were the reason, which did not appear to be connected with the consumption of the lozenge. Compliance in the study was good with only one of the subjects reporting that she used less than the four recommended products per day.

## Discussion

Our results demonstrated a LGG-BB-12-induced decrease in the plaque index, which promoted a decrease in the gingival index. To our knowledge, this is the first study to demonstrate such an effect on gingival health of this probiotic combination. Importantly, the result was obtained in healthy subjects.

Most studies present the suggestion that probiotics could control plaque by decreasing periodontopathogens or MS [24, 27]. Recently, *L. reuteri* was shown to decrease counts of selected periodontopathogens both with and without a clinical impact [40, 41]. No studies have connected LGG and/or BB-12 consumption with decreases in counts of periodontopathogens. Our subjects possessed a microbiota typical for healthy subjects [42]. The subjects did not harbor any major periodontopathogens at baseline; thus, a decrease in PI and GI could not be associated to these microorganisms.

In addition to the HOMIM analyses of the microbiota, we also cultured the saliva samples since a multitude of reference data exist for plate-cultured MS and lactobacilli (LB). No study-induced effects on salivary MS counts were found in the probiotic group. Earlier studies detected decreases or no changes in MS in association with LGG consumption [19, 28, 29]. BB-12 consumption has been associated with decreases in MS counts [27], but this effect could not be detected in our study. The method we used to culture the LB could detect salivary LB as well as the LGG of the probiotic product, but not BB-12. The consumption of the combination of LGG and BB-12 had no effect on the salivary LB counts. As for LGG, the result is in agreement with our earlier study [28]. Clinical studies have shown for both LGG [15, 17] and BB-12 [17, 18] poor retention to the oral cavity. Thus, even though we did not determine BB-12, it should not have been present in the saliva samples. The negative result of the HOMIM supports this consideration.

The sugars of hydrolysed plaque reflect the polysaccharide content of plaque and the proteins the amount of microbes in the plaque. Polysaccharides of plaque have been used as indicators of the virulence of plaque, a.o. adhesivity [36, 37]. In our study, the polysaccharide content of the plaque did not change. This suggests that the decreases found for PI and GI in the probiotic group were not associated with changes in the polysaccharide contents of the plaques.

Immune modulation of the host could be a possible explanation for the improved periodontal status in the probiotic group, since no change in the oral microbiota or the adhesion properties of plaque could be demonstrated. Earlier, consumption of *L. reuteri* reduced pro-inflammatory cytokines in the crevicular fluid of adults with gingival inflammation [43]. This finding may reflect a local effect oral immune responses, since *L. reuteri* is effective in treating diarrhea but does not improve the immune response of the host [2]. However, LGG and BB-12 have even in short-term use shown beneficial effects on the immune responses of children and adults [44–46], which should be reflected thus also in the composition of the crevicular fluid. LGG and *B. lactis* were both evaluated in a consensus opinion to have the effectiveness of grade A in improving the immune response of the host [2]. The combination of LGG and BB-12 appears to be even more effective in this respect compared to LGG or BB-12 alone [6–8].

Taken together, the combination of LGG and BB-12 improved periodontal health in healthy subjects, without affecting the composition of the oral microbiota or adhesion properties of plaque.
